# International Delphi consensus on the management of percutaneous choleystostomy in acute cholecystitis (E-AHPBA, ANS, WSES societies)

**DOI:** 10.1186/s13017-024-00561-8

**Published:** 2024-10-12

**Authors:** José M. Ramia, Mario Serradilla-Martín, Celia Villodre, Juan J. Rubio, Fernando Rotellar, Ajith K. Siriwardena, Go Wakabayashi, Fausto Catena, PERCHODEL Collaborative Study Group, Tomoyuki Abe, Yuta Abe, Fikri Abu-Zidan, Cándido F. Alcázar López, Ryusuke Amemiya, Bodil Andersson, Luca Ansaloni, Anita Balakrishnan, Zsolt J. Balogh, Silvia Carbonell-Morote, Ahmet Coker, Dimitrios Damaskos, Belinda De Simone, Jonh Devar, Isabella Frigerio, Yusuke Fujita, Sigheo Hayatsu, Shutaro Hori, Sho Ibuki, Noriaki Kameyama, Youichi Kawano, Andrew Kirkpatrick, Jorg Kleeff, Yoram Kluger, Rifat Latifi, Santiago Lopez-Ben, Giuseppe Malleo, Yuki Masuda, Takuya Minagawa, Kohei Mishima, Ryohei Miyata, Ernest Moore, Ryo Nishiyama, Yusuke Ome, Junichi Saito, Alejandro Serrablo, Masaya Shito, Kjetil Soreide, Oliver Strobel, Michael Sugrue, Keiichi Suzuki, Yutaka Takigawa, Moriaki Tomikawa, Hidejiro Urakami, Carlo Vallicelli, Taiga Wakawayashi, Dieter Weber

**Affiliations:** 1 Department of Surgery, Hospital General Universitario Dr. Balmis, C/Sol Naciente 8, 16D 3016 Alicante, Spain; 2 ISABIAL, Alicante, Spain; 3 University Miguel Hernandez, Alicante, Spain; 4 Department of Surgery, Hospital Universitario Virgen de Las Nieves, Granada, Spain; 5 Instituto de Investigación Biosanitaria Ibs.GRANADA, Granada, Spain; 6 Department of Surgery, School of Medicine, University of Granada, Granada, Spain; 7 HPB and Liver Transplantation Unit, Department of Surgery, University Clinic, Universidad de Navarra, Institute of Health Research of Navarra (IdisNA), Pamplona, Spain; 8 Regional Hepato-Pancreato-Biliary Unit, Manchester Royal Infirmary, Manchester, UK; 9 Department of Surgery, Ageo Central General Hospital, Ageo, Japan; 10 Emergency and Trauma Surgery, Bufalini Hospital, Cesena, Italy

**Keywords:** Acute cholecystitis, Percutaneous cholecystectomy, Delphi, Consensus, Outcomes

## Abstract

**Background:**

There has been a progressive increase in the use of percutaneous cholecystostomy (PC) in acute cholecystitis (AC) over the last decades due to population aging, and the support of guidelines (Tokyo Guidelines (TG), World Society of Emergency Surgery (WSES) Guidelines) as a valid therapeutical option. However, there are many unanswered questions about the management of PCs. An international consensus on indications and PC management using Delphi methodology with contributions from experts from three surgical societies (EAHPBA, ANS, WSES) have been performed.

**Methods:**

A two-round Delphi consensus, which included 27 questions, was sent to key opinion leaders in AC. Participants were asked to indicate their ‘agreement/disagreement’ using a 5-point Likert scale. Survey items with less than 70% consensus were excluded from the second round. For inclusion in the final recommendations, each survey item had to have reached a group consensus (≥ 70% agreement) by the end of the two survey rounds.

**Results:**

54 completed both rounds (82% of invitees). Six questions got > 70% and are included in consensus recommendations: In patients with acute cholecystitis, when there is a clear indication of PC, it is not necessary to wait 48 h to be carried out; Surgery is the first therapeutic option for the TG grade II acute cholecystitis in a patient suitable for surgery; Before PC removal a cholangiography should be done; There is no indication for PC in Tokyo Guidelines (TG) grade I patients; Transhepatic approach is the route of choice for PC; and after PC, laparoscopic cholecystectomy is the preferred approach (93.1%).

**Conclusions:**

Only six statements about PC management after AC got an international consensus. An international guideline about the management of PCs are necessary.

## Background

Acute cholecystitis (AC) accounts for 30% of emergency admissions to general surgery departments and is the second most frequent cause of complicated intra-abdominal infection [1]. Currently, laparoscopic cholecystectomy (LC) is the gold standard in the treatment of AC. However, in patients with high surgical risk, comorbidities, or advanced age, LC is associated with high rates of morbidity (31%), and postoperative mortality (4%); much higher than those obtained in patients with low surgical risk [2].

In patients with a level of surgical risk that outweighs the possible benefits of surgery, non-surgical treatments have become widespread [3, 4]. These treatments, including the most frequently used alternative to surgery, percutaneous cholecystostomy (PC), play a crucial role in improving patient outcomes. PC consists of the percutaneous puncture of the gallbladder and the placement of a drainage catheter, usually performed in very ill patients as a bridge to a delayed surgical procedure [5].In some countries, an increase in the use of PC over the last decades has been observed due to two main factors [6]: Population aging, which increases the number of patients with high surgical risk, and the publication of the Tokyo Guidelines (TG) and the World Society of Emergency Surgery (WSES) Guidelines. These guidelines devised to standardize the diagnosis, management, and treatment of AC, and recommended the use of PC in selected groups of patients [3, 4, 7, 8]. Recently, however, the utility of PC versus LC in patients with high surgical risk has been questioned, and it has been suggested that PC may be over-used [4, 9].

The theoretical advantages offered by PC are the rapid resolution of sepsis and the optimal preparation of the patient for elective LC [5, 9]. Its main drawback is the possibility of recurrence of AC or other biliary events while awaiting LC. Many questions, indications, and management of PCs are unanswered.

The Delphi method is a well-established approach for answering a research question by identifying a consensus view among subject experts. It allows for reflection among participants, who can reconsider their opinions based on the anonymized views of their peers [10–13]. First, available evidence should be reviewed to develop the Delphi consensus questionnaire. Finally, a Delphi process is delivered to formulate these guidance and recommendations [11–14]. This study defines an international consensus on indications and PC management using Delphi methodology with contributions from experts from three surgical societies. This method lets us know the opinion of a group of experts, but only randomized control trials could assure us that Delphi’s statements are adequate.

## Methods

This Delphi consensus consisted of four phases, each informing the subsequent phase. The study did not require approval by an Ethics committee because there was no contact with patients and all expert participation was on a voluntary basis.

### Phase 1: evidence acquisition

A non-systematic review was undertaken (JR and JJR) to acquire the most new and relevant information on the use of PCs in AC. using the keywords “cholecystostomy” and “acute cholecystitis”“ (years 2018–2023) in Pubmed, EMBASE, Cochrane and SCielo databases.

### Phase 2: expert panel virtual discussion

A core expert committee of six experts (JR, FC, FR, MSM, AKS, GW) on AC was invited and agreed to participate. The members represented three societies (EAHPBA (European-African Hepato-Pancreato-Biliary Association), the ANS Japanese Group led by GW, and WSES [World Society of Emergency Surgery]). This panel of experts discussed the themes identified in Phase 1 over structured virtual discussion sessions. Finally, the Core group included 27 questions in the Delphi and asked experts (Table 1).

**Table 1:** Results of round 1 of the Delphi study

Item	Median	1	2	3	4	5	4 + 5
In patients with AC when there is a clear indication of PC it is not necessary to wait 48 h to be carried out	4	3.4	3.4	1.7	50.0	41.4	**91.4**
There is no indication for PC in Tokyo Guidelines (TG) grade I patients	4	3.4	13.8	10.3	41.4	31.0	*72.4*
PC is the first therapeutic option for the TG grade II AC in a patient suitable for surgery	2	39.7	41.4	10.3	6.9	1.7	8.6
Surgery is the first therapeutic option for the TG grade II AC in a patient suitable for surgery	4	0.0	3.4	5.2	43.1	48.3	**91.4**
PC is the first therapeutic option for the TG grade III AC in a patient suitable for surgery	2.5	17.2	32.8	13.8	32.8	3.4	36.2
Surgery is the first therapeutic option for the TG grade III AC in a patient suitable for surgery	4	3.4	20.7	22.4	36.2	17.2	53.4
PC is the first therapeutic option for TG grade II/III AC with suspected common bile duct stones	2	19.0	36.2	19.0	20.7	5.2	25.9
PC is the first therapeutic option for TG grade II/III AC and severe local inflammation that implies a very difficult cholecystectomy	4	6.9	20.7	15.5	43.1	13.8	56.9
PC should be performed to all ASA III patients with AC	2.5	13.8	36.2	27.6	22.4	0.0	22.4
PC should be performed to all ASA IV patients with AC	4	6.9	13.8	17.2	51.7	10.3	62.1
PC should be performed to all patients with a septic shock in any AC grade	4	10.3	19.0	12.1	43.1	15.5	58.6
PC should be performed in patients with > 72 h symptoms	2	13.8	39.7	29.3	15.5	1.7	17.2
In a patients non-suitable for surgery. a scheduled endoscopic cholecystoduodenostomy should be performed to avoid recurrent cholecystitis	3	5.2	25.9	29.3	36.2	3.4	39.7
Transhepatic approach is the route of choice for PC	4	6.9	1.7	19.0	32.8	39.7	*72.4*
When clinical and analytical improvement occur PC could be closed	4	6.9	12.1	17.2	55.2	8.6	63.8
PC should stay always 6 weeks open	2	25.9	44.8	15.5	12.1	1.7	13.8
Before PC removal a cholangiography should be done	4	0.0	12.1	8.6	44.8	34.5	*79.3*
If contrast does not pass to common bile duct PC should be kept in place and open until cholecystectomy	4	0.0	22.4	10.3	46.6	20.7	67.2
After cholangiography PC will stay closed for 6 weeks	2	15.5	48.3	25.9	8.6	1.7	10.3
If there is a normal cholangiogram. PC will be closed and retired in 48 h if there is not a clinical/analytical worsening	4	3.4	8.6	17.2	56.9	13.8	*70.7*
If after PC removal a new AC episode occurs. a new PC should be performed	3	6.9	19.0	50.0	20.7	3.4	24.1
In very fragile patients never suitable for surgery and endoscopic cholecystoduodenostomy should be scheduled for recurrent AC prevention	3	5.2	25.9	25.9	36.2	6.9	43.1
PC increases the difficulty of cholecystectomy by laparoscopic approach	2	20.7	44.8	12.1	19.0	3.4	22.4
PC increases the difficulty of cholecystectomy by open approach	2	27.6	43.1	13.8	12.1	3.4	15.5
After PC open cholecystectomy is the preferred approach	1	55.2	41.4	1.7	1.7	0.0	1.7
After PC laparoscopic cholecystectomy is the preferred approach	4	1.7	0.0	5.2	56.9	36.2	**93.1**
After PC. cholecystectomy should be performed after 8 weeks if patient medical condition is adequate	3	13.8	17.2	22.4	41.4	5.2	46.6

Showing medians and percentages of responses on the Likert scale for each of the items

### Phase 3: Delphi process

Following phase 2, Delphi methodology was used to quantify consensus in the participating Group. Delphi was performed electronically using (Google Forms®, Mountain View, CA, USA). The language used to carry out the questionnaire was English. The Delphi was distributed to all phase 2 Core group members and 60 key opinion leaders in AC worldwide, with sound theoretical knowledge of the area, and a high degree of practical expertise acknowledged by their peers in the field.

Each expert received a link to an online questionnaire via email. All participants were asked to propose additional criteria or reflections they considered necessary in free text fields only in the first round. All responses were treated anonymously. Only the initials of each participant were recorded, along with their hospital and country of origin, to avoid duplication of questionnaires. A round electronic consensus exercise was then conducted [10–14] (Round 1: 11/January/2024 to 11/February/2024; Round 2: 1/April/2024 to 15/May/2024). A first email was sent to the experts at the beginning of each round, followed by two weekly reminders. An interval of three weeks between rounds was scheduled to analyze the results and prepare for the next round. 

Participants were asked to indicate their ‘agreement/disagreement’ with the proposed parameters using the questionnaire comprising questions to be answered on a 5-point Likert scale: 1: “totally disagree,” 2: “disagree,” 3: “neither agree nor disagree, “4: “agree,” and 5: “totally agree” [13]. Survey items with less than 70% consensus were excluded from the second survey round, with the consensus threshold achieved disseminated to all participants. For inclusion in the final recommendations, each survey item had to have reached a group consensus (≥ 70% agreement) by the end of the two survey rounds. Items that did not achieve consensus were also discussed in phase 4.

To define the degree of agreement, the following criteria were used:

- “Unanimity”: when 100% of the participants gave the same response on the Likert scale.
- “Agreement”: when ≥ 80% of the participants agreed.
- “Majority”: when ≥ 70% agreed.
- “Discrepancy”: when < 70% agreed.

### Phase 4: generation of recommendations

The Core group summarized and reported the recommendations within this manuscript based on the consensus results of the Delphi process.

### Statistical analysis

Only complete questionnaires were considered, and each round’s response rate was recorded. The results for each round were expressed as the percentage of responders for each answer option (from 1 to 5), together with the median and range for each item. The analysis assessed the responses in the online platform’s database. Descriptive statistics for categorical variables were reported as numbers and percentages, while continuous variables were reported as means and standard deviations (SD). The delta of changes between rounds 1 and 2 was calculated. The p value was calculated using the chi-square test with Yates correction for percentages of responses (4 + 5), and U Mann- Whitney-Wilcoxon Test for medians of each category.

## Results

**Fig. 1:**
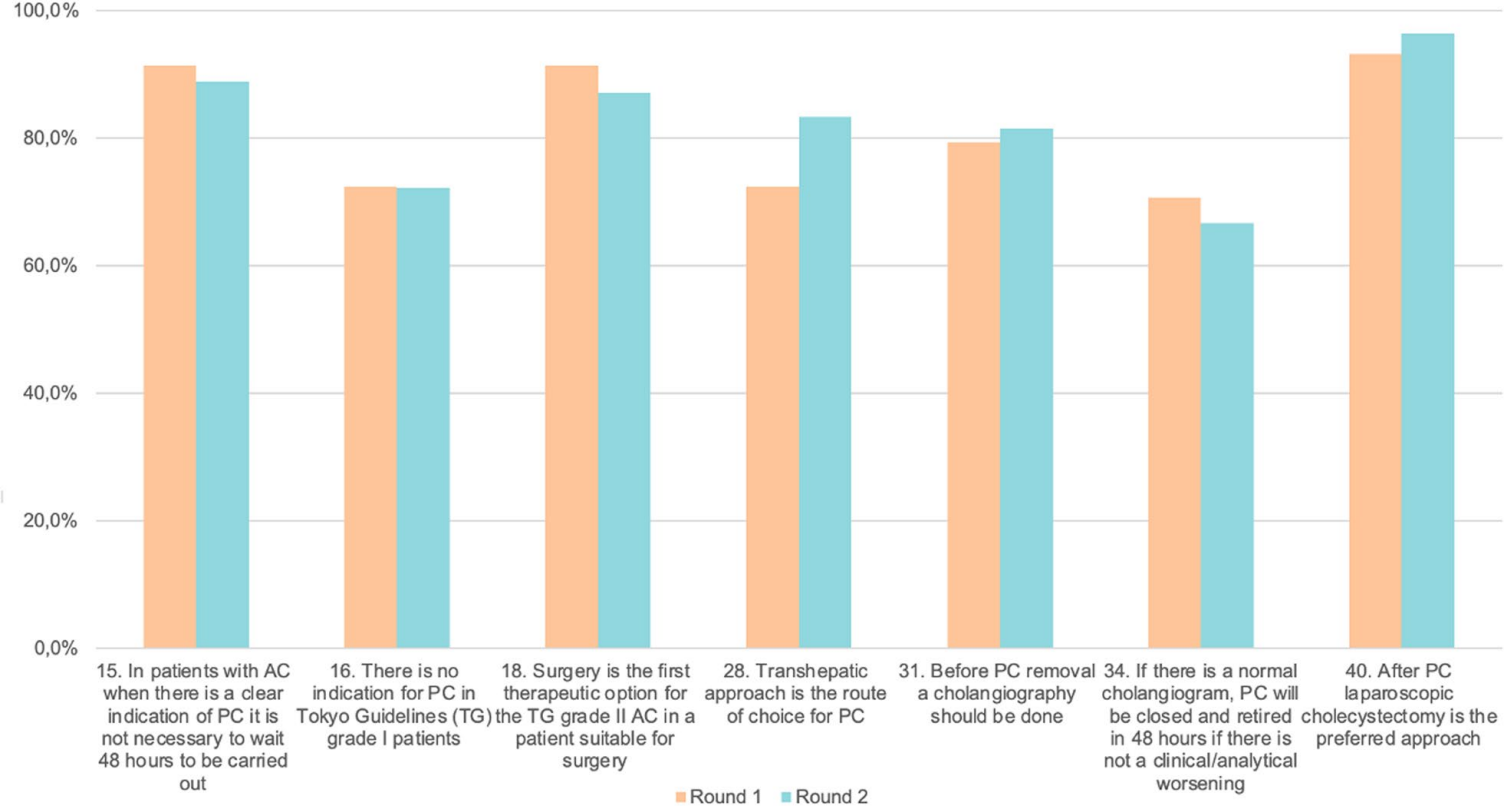
Questions that passed 70% consensus

The survey was sent to 66 surgeons (20 by Society) plus the Core group. There were 58 responders in the first round, and 54 completed both rounds (82% of invitees) and these were the answers included in the analysis. Surgeons were from: Japan (22), Spain (7), Italy (7), United Kingdom (3), Australia, France, and USA (2), Austria, Germany, Ireland, Israel, Norway, South Africa, Sweden, Turkey, and the United Arab Emirates (1). The median age was 50 years (IQR: 43–56). Thirty-two surgeons work in a public academic hospital, seven in a public non-academic, thirteen in a private academic hospital, and two in a private non-academic center. The median of beds in the hospital was 675 (IQR 400–970). The hospital has a 24-h PC available in 48/54 (88.8%), an ERCP 24-h in 40/54 (74.1%), EUS 24-h in 19/54 (35.2%), and a surgeon on call in all centers (Fig. 1).

In the first round, only seven questions reached 70%, adding agree and totally agree on answers (Table 1) and passing to 2nd round. The questions were: **Question 1:** In patients with acute cholecystitis, when there is a clear indication of PC it is not necessary to wait 48 h to be carried out (91.4%); **Question 2:** Surgery is the first therapeutic option for the TG grade II acute cholecystitis in a patient suitable for surgery (91.4%); **Question 3:** Before PC removal a cholangiography should be done (79.3%); **Question 4:** There is no indication for PC in Tokyo Guidelines (TG) grade I patients (72.4%); **Question 5:** Transhepatic approach is the route of choice for PC (72.4%); **Question 6:** If there is a normal cholangiogram, PC will be closed and retired in 48 h if there is no clinical/analytical worsening (70,7%); **Question 7:** After PC, laparoscopic cholecystectomy is the preferred approach (93.1%).

In the second round, six of seven questions reached > 70%. The question, “If there is a normal cholangiogram, PC will be closed and retired in 48 h if there is not a clinical/analytical worsening,” received only 67.27% of agree plus totally agree, so it was not included in the final recommendations (Tables 2 and 3). The Delta between both rounds was calculated (Table 4).

**Table 2:** Results of round 2 of the Delphi study

		Median	1 (%)	2 (%)	3 (%)	4 (%)	5 (%)	*P* value
In patients with acute cholecystitis when there is a clear indication of PC it is not necessary to wait 48 h to be carried out	Round 2	5	5.56	3.70	1.85	27.78	61.11	
	Δ		2.16	0.30	0.15	− 22.22	19.71	0.095
There is no indication for PC in Tokyo Guidelines (TG) grade I patients	Round 2	4	0.00	16.67	11.11	48.15	24.07	
	Δ		− 3.40	2.87	0.81	6.75	− 6.93	0.669
Surgery is the first therapeutic option for the TG grade II acute cholecystitis in a patient suitable for surgery	Round 2	5	0.00	3.70	9.26	27.78	59.26	
	Δ		0.00	0.30	4.06	− 15.32	10.96	0.420
Transhepatic approach is the route of choice for PC	Round 2	4.5	1.85	1.85	12.96	33.33	50.00	
	Δ		− 5.05	0.15	− 6.04	0.53	10.30	0.154
Before PC removal a cholangiography should be done	Round 2	4	1.85	5.56	11.11	38.89	42.59	
	Δ		1.85	− 6.54	2.51	− 5.91	8.09	0.415
If there is a normal cholangiogram. PC will be closed and retired in 48 h if there is not a clinical/analytical worsening	Round 2	4	1.85	16.67	14.81	48.15	18.52	
	Δ		− 1.55	8.07	− 2.39	− 8.75	4.72	0.912
After PC laparoscopic cholecystectomy is the preferred approach	Round 2	4.5	0.00	0.00	3.70	46.30	50.00	
	Δ		− 1.70	0.00	− 1.50	− 10.60	13.80	0.123

Medians and percentages for responses on the Likert scale for each of the 7 items obtained in round 1 and included in the form sent out in round 2. Δ is calculated as round 2—round 1. The *p* value was calculated using Mann–Whitney-Wilcoxon Test

**Table 3:** Items included in the questionnaire sent out in round 2 together with the medians and percentages of responses 4 + 5 (agree and totally agree) for each one

	Median	4 + 5 (%)
In patients with acute cholecystitis when there is a clear indication of PC it is not necessary to wait 48 h to be carried out	5	88.9
There is no indication for PC in Tokyo Guidelines (TG) grade I patients	4	72.2
Surgery is the first therapeutic option for the TG grade II acute cholecystitis in a patient suitable for surgery	5	87.0
Transhepatic approach is the route of choice for PC	4.5	83.4
Before PC removal a cholangiography should be done	4	81.5
If there is a normal cholangiogram. PC will be closed and retired in 48 h if there is not a clinical/analytical worsening	4	*66.7*
After PC laparoscopic cholecystectomy is the preferred approach	4.5	96.3

**Table 4:** Likert scale on rounds 1 and 2 for the 7 items selected after round 1

	Round 1 (%)	Round 2 (%)	Delta	*P* value
In patients with acute cholecystitis when there is a clear indication of PC it is not necessary to wait 48 h to be carried out	91.4	88.9	− 2.5	0.266
There is no indication for PC in Tokyo Guidelines (TG) grade I patients	72.4	72.2	− 0.2	0.096
Surgery is the first therapeutic option for the TG grade II acute cholecystitis in a patient suitable for surgery	91.4	87.0	− 4.4	0.642
Transhepatic approach is the route of choice for PC	72.4	83.4	11.0	0.007
Before PC removal a cholangiography should be done	79.3	81.5	2.2	0.017
If there is a normal cholangiogram. PC will be closed and retired in 48 h if there is not a clinical/analytical worsening	70.7	66.7	− 4.0	0.523
After PC laparoscopic cholecystectomy is the preferred approach	93.1	96.3	3.2	0.063

Δ is calculated as round 2 – round 1. The p value calculated using chi-square test with Yates correction

## Discussion

In Delphi study, there was only a consensus on six of twenty-seven questions covering all aspects of PC management in AC. Three of them, on the indication: no need to wait 48 h if the indication is evident from the first moment, and PC should not be performed in AC grades I and II of TG. The other three focused on technique: the best route for performing PC is transhepatic, and before removing the PC a cholangiography through catheter must be performed, and late management: laparoscopic cholecystectomy is the treatment of choice, even if PC has been performed.

PC was first applied by Radder et al. in 1980 [14]. PC is a procedure with a high technical success rate and high disposable (88% of responders had PC 24/7). It is safe and associated with low morbidity. A systematic review reported a complication rate of 14%, and allows rapid control of the focus of infection and rehabilitation of the patients for scheduled surgery [14–17]. But PC has some limitations: patient discomfort, around 25% of patients treated with PC required the placement of a new PC, readmission rates are high (30%). PC could solve the initial clinical scenario, but biliary lithiasis, the source of the problem, is not solved [18].

Now, a comparison with previous publications on the questions included and approved in Delphi is done.

- *Performing PC as soon as decision is taken without waiting 48 h of clinical evaluation* Some manuscripts confirm that early PC reduces hospital stay and slows the progression of the inflammatory condition [19]. WSES guidelines recommended to wait 24–48 h in patients not suitable for surgery and treating with antibiotics and close observation [4]. In TG guidelines an early/urgent PC is recommended but no precise data about timing is included [8]. So, this could be the first clinical outstanding recommendation of this Delphi, the PC should be done, if it is indicated, as soon as possible.
- *PC in different grades of AC and specific clinical scenarios* There is a clear consensus that patients with AC TG Grade I and II should be operated on [4, 8, 20]. For Grade III patients, PC should not be considered the first option if the surgeon finds that patient is fit for surgery [4, 8]. The CHOCOLATE randomized trial comparing high surgical risk patients treated with PC or LC did not find differences in mortality rates. But they observed higher rates of complications, reoperations, and recurrence of biliary pathology in PC patients [21]. So, the main accepted indication of PC is patients with AC who are unable to undergo surgery due to comorbidities (unfit for surgery and/or shock or severe sepsis) [4, 8, 19]. Some manuscripts and guidelines also admit other indications for PC, such as AC > 72 h, marked local inflammation, or leukocyte count > 18,000 L/mm3 [3, 4, 14]. In this Delphi were questions about these extra indications for PC, and the recommendation was not to use PC based on fragility (ASA III and IV), suspected common bile duct stones, or difficult cholecystectomy. So, the Delphi answers have a great adherence to WSES and TG guidelines and considered PC the best options in unfit or extremely sick patients, not considering PC a good option for extra scenarios.
- *Clinical management of PC* There has yet to be an international consensus about the management of PC [4, 8]. One of the most controversial issues is the duration of the drain placement. Some authors recommend keeping it in place until surgery or at least six weeks since early removal is associated with complications. Others suggest its withdrawal when the AC has resolved [21]. The answers show that 69% of the responders disagree or totally disagree with a policy of 6 weeks open. In the first round, the question about if cholangiogram is normal, PC will be closed and retired in 48 h if there is not a clinical/analytical worsening pass the cutoff of 70% in first round but not in the second round. There is a clear consensus that best route for performing PC is transhepatic, and before removing the PC a cholangiography through catheter must be performed. Management of PC is not usually included in guidelines but would be very interesting for avoiding variability [4, 8]. But it could not be recommended a clear timing of PC [22].
- *Cholecystectomy after PC* In the literature, the rate of cholecystectomy after PC varies from 36 to 57% [14, 23–30]. There are no reports providing quality scientific evidence on the best timing for surgery after PC. TG and WSES did not perform clear indications about this topic [4, 8, 23–31]. Total healthcare costs are lower in patients who undergo cholecystectomy in the first two months after PC. A study comparing early cholecystectomy (0–8 weeks) versus late cholecystectomy (> 8 weeks) found that the early cholecystectomy group had a higher risk of complications and longer hospital stay. A 2021 systematic review concluded that the interval of 9–10 weeks after PC is the optimal time for cholecystectomy. Finally, a 2022 meta-analysis comparing cholecystectomy during the first 30 days and at a later did not find differences in the clinical results [14, 23–30]. Other point that has not been demonstrated is that cholecystectomy is difficult after PC, so some surgeons recommend open surgery after PC. In the Delphi, there was a clear consensus about laparoscopic approach should be performed.

Although this is not the objective of this manuscript, there is another therapeutical option for patients with clear PC indication. Endoscopic Ultrasound-Guided Gallbladder Drainage (EUS-GBD) has also been proven to be a feasible technique for treating AC unfit for surgery with fewer adverse events and a lower reintervention rate than PC [32, 33]. The advantages of EUS-GBD vs PC include internalization of bile, obviating the risk of recurrent cholecystitis following PC removal and the risk of bleeding, and being associated with less post-procedural pain [31, 32]. However, the 24-h availability of EUS is less than that of PC; for example, the difference is evident in the centers where the panel members work (88 vs. 35%), and the need for advanced endoscopic expertise not disposable in all centers should be considered.

The limitations of this study are intrinsic to any Delphi Study, and the strength is that panelists are from all over the world, which decreased the bias of being from only one country with a specific health system.

## Conclusions

In conclusion, only six statements about PC management after AC got an international consensus. Clear guidelines about the management of PC are necessary.

## Data Availability

The datasets used and/or analyzed during the current study are available from the corresponding author on reasonable request for 10 years
